# The science of feeling and emotion: From past to present

**DOI:** 10.1192/j.eurpsy.2023.1955

**Published:** 2023-07-19

**Authors:** S. E. Ilgin

**Affiliations:** 1 Neuroscience, Yeditepe University, Istanbul, Türkiye

## Abstract

**Introduction:**

As the founder of modern psychology as a discipline Wilhelm Wundt came up with the theory of tridimensional feeling (Wundt. Grundriss der Psychologie 1922; Leipzig), which has evolved over time with different theories and is thought to be essential for human survival. A feeling is the conscious awareness of the emotion itself. Feelings are personal and biographical, emotions are social, and affects are impersonal.

**Objectives:**

We intend to understand how emotions can be explained through theories since the beginning of the modern psychology.

**Methods:**

We performed a review of the published literature on the subject using Pubmed. We conducted a search using ‘feeling’ , ‘emotion’ and ‘affect’ as keywords.

**Results:**

Although there are many theories on emotions they conclude that for centuries emotions have various functions and they help us survive. In order to explain this we can make use of biopsychosocial perspectives.The history of study of feeling began with Wundt’s theory of tridimensional feeling and later on different theories such as structuralism, functionalism, evolutionary perspective, behaviorism and nowadays most famous theory neuropsychoanalysis were proposed. Affect can be described as the individual’s ability to participate in stimuli, events, memories and thoughts with an emotional response, on the other hand feelings are the subjective complements of sensations but do not originate necessarily from a sense organ. Moreover, emotion is the reflection of a feeling.

**Conclusions:**

Based on our research, we conclude that for almost over a century there are still theories being developed on feelings and in this matter biopsychosocial perspective has a critical role on its advancement. Are emotions just telltale signs of homeostasis, as Damasio points out? (Damasio & Carvalho. Nat. Rev. Neurosci 2013; 14(2), 143–152.) Apart from their physiological functions, can there be emotions that were once experienced and then suppressed and pushed into the unconscious? If we explain the unconscious only with the functioning of procedural memory in accordance with the current findings of neuroscience, then some changes are needed in our understanding of psychotherapy and especially transference. Because “is the thing that helps change in psychotherapy, the expression of an idea, the verbalization of the experiences, or an emotional/affective exchange between the psychotherapist and the patient?” We must find the answer to the question. It is hoped that neuroscience in general and neuropsychoanalysis in particular will reach new findings and explanations on these issues in the near future.

**Disclosure of Interest:**

None Declared

